# 66 Evaluation of an Organic Bioethanol Based Hand Rub: User Acceptance and Skin Tolerability Study

**DOI:** 10.1017/ash.2026.10497

**Published:** 2026-06-23

**Authors:** Abdoul Rahamane Bako Bagassa, Katie Thure, Glodi Mutamba, Callyn Wren, Christopher Evans, Becky Meyer

**Affiliations:** 1 Tennessee Department of Health; 2 TN Department of Health

## Abstract

**Background:** Inappropriate antibiotic prescribing drives unnecessary healthcare expenditures and antimicrobial resistance. In 2022, the U.S. issued over 200 million outpatient antibiotic prescriptions, with 30% considered unnecessary. Although the clinical effects of overprescribing are well-known, its economic impact at the state level is less studied. This study estimated the financial burden of unnecessary antibiotic use in Tennessee in 2022 and examined differences based on age, rural or urban settings, and antibiotic class. Methods The IQVIA Medical Claims and Longitudinal Prescription Claims databases were used to perform a cross-sectional study evaluating the cost burden of unnecessary outpatient antibiotics dispensed from retail pharmacies in Tennessee from January 1–December 31, 2022. Antibiotic prescriptions were categorized into three tiers based on appropriateness using ICD-10 codes, with only Tier 3 (unnecessary) prescriptions included in the analysis. Prescriptions were aggregated by National Drug Code (NDC). Minimum, maximum, and average unit costs were obtained from Lexidrug-UpToDate and multiplied by the dispensed supply to estimate prescription-level costs. These costs were aggregated and stratified by age, residence, and antibiotic class. Results Conclusion Unnecessary antibiotic prescribing imposes a substantial economic burden, with annual costs reaching up to $245 million and notable out-of-pocket expenses for patients, disproportionately affecting older adults, rural residents, and certain antibiotic classes. Targeted stewardship interventions focusing on high-cost medications and prescribing patterns can reduce costs and antimicrobial resistance. Future research should evaluate tailored interventions and policies to promote proper prescribing across diverse demographic and geographic settings.

